# Sex differences in the branching position of the nerve to the abductor digiti minimi muscle: an anatomical study of cadavers

**DOI:** 10.1186/s13047-015-0077-6

**Published:** 2015-06-10

**Authors:** Daisuke Mizuno, Munekazu Naito, Shogo Hayashi, Yusuke Ohmichi, Mika Ohmichi, Takashi Nakano

**Affiliations:** Department of Anatomy, Aichi Medical University School of Medicine, Nagakute, Aichi 480-1195 Japan; Department of Anatomy, Tokyo Medical University School of Medicine, Shinjuku-ku, Tokyo Japan

**Keywords:** Heel pain, Sex difference, Abductor digiti minimi muscle, Lateral plantar nerve, Posterior tibial nerve, Cadaver dissection

## Abstract

**Background:**

The nerve to the abductor digiti minimi muscle (ADMM nerve) is the first branch of the lateral plantar nerve or originates directly from the posterior tibial nerve. Damage to the ADMM nerve is a cause of heel pain and eventually results in ADMM atrophy. It is known that ADMM atrophy occurs more often in females than in males, and the reason remains unclear. This study aimed to explore sex differences in the branching pattern, position, and angle of the ADMM nerve.

**Methods:**

Forty-two cadavers (20 males, 22 females) were dissected at Aichi Medical University between 2011 and 2015. Cases of foot deformity or atrophy were excluded and 67 ft (30 male, 37 female) were examined to assess the branching pattern, position, and angle of the ADMM nerve.

**Results:**

The branching positions of the ADMM nerve were superior to the malleolar–calcaneal axis (MCA) in 37 ft (55 %), on the MCA in 10 ft (15 %), and inferior to the MCA in 20 ft (30 %). There was no case among male feet in which the ADMM nerve branched inferior to the MCA, whereas this pattern was observed in 19 of 37 female feet (51 %). The branching position of the ADMM nerve was significantly closer to the MCA in female feet than in male feet. There were no significant sex differences in the branching pattern and angle of the ADMM nerve.

**Conclusions:**

The ADMM nerve sometimes branches off inferior to the MCA in females, but not in males. This difference may be the reason for the more frequent occurrence of ADMM atrophy in females than in males.

## Background

There are various causes of heel pain, including plantar fasciitis, plantar fat pad disorders, seronegative spondyloarthropathies, and calcaneal stress fractures. One of the more difficult-to-diagnose causes of heel pain is damage to the nerve to the abductor digiti minimi muscle (hereafter referred to as the ADMM nerve) [1]. It is known that damage to the ADMM nerve eventually results in ADMM atrophy. In a retrospective study, 10 of 476 patients (2.1 %) undergoing magnetic resonance imaging (MRI) of the foot and ankle had ADMM atrophy, suggesting that ADMM atrophy is helpful for confirming the diagnosis of damage to the ADMM nerve [2]. In that study, 9 of the 10 patients (90 %) with ADMM atrophy were females [2]. Furthermore, a prospective study of MRI of ankles and feet demonstrated that 38 of 602 patients (6.3 %) had selective fatty atrophy of the ADMM, with 29 of the 38 patients (76 %) being female [3]. This atrophy was believed to be associated with obesity, presence of a plantar heel spur, and complex and multifocal aberrations in the hindfoot and ankle [2]. However, it remains unclear why ADMM atrophy occurs more often in females than in males.

The nerve bundle innervating the ADMM (i.e., ADMM nerve) has been referred to by several names, including the inferior calcaneal nerve, deep calcaneal nerve, and Baxter’s nerve [4, 5]. The ADMM nerve arises either as the first branch of the lateral plantar nerve or directly from the posterior tibial nerve, and runs in the medial-to-lateral direction between the abductor hallucis muscle and the medial calcaneal tuberosity [6–9]. The ADMM nerve is a mixed sensory and motor nerve that supplies motor branches to the ADMM and, occasionally, supplied the flexor digitorum brevis and quadratus plantae muscles and sensory branches to the calcaneal periosteum and the long plantar ligament [8]. The ADMM nerve runs plantar from its origin, at a significant depth in relation to the abductor halluces muscle. The ADMM nerve changes direction from vertical to horizontal at the inferior margin of the abductor hallucis. As it courses laterally, the nerve lies anterior to the medial process of the calcaneal tuberosity between the quadratus plantae dorsally and the plantar fascia and flexor digitorum brevis muscle in its plantar aspect. The ADMM nerve continues laterally and penetrates the proximal part of the ADMM. A more recent study showed that the ADMM nerve is actually located more posteriorly at an average 5.5 mm anterior to the medial process of the calcaneal tuberosity [10]. However, sex differences in the ADMM nerve are unknown. The aim of this study was to explore sex differences in the branching pattern, position, and angle of the ADMM nerve in cadaveric feet.

## Methods

Forty-two cadavers (males *n* = 20, females *n* = 22) were examined in this study. The cadavers were donated to Aichi Medical University between 2011 and 2015. Before they died, the donors signed documents agreeing to body donation and its use for clinical studies. The format of the document is within the expectation of the Japanese law “Act on Body Donation for Medical and Dental Education.” The average age of the cadavers was 83.2 ± 6.5 years (range, 70–97 years; males, 84 ± 6.4 years; females, 83 ± 7 years). Cases of foot deformity or atrophy were excluded from the analysis, and 67 ft (30 male, 37 female) were dissected to assess the branching pattern, position, and angle of the ADMM nerve. After finding the tibial nerve, the flexor retinaculum was cut to identify the posterior tibial nerve and lateral plantar nerve. The nerves were followed distally to identify the branching positions of the ADMM nerve. The cutaneous branch of the ADMM nerve was also identified.

**Fig. 1:**
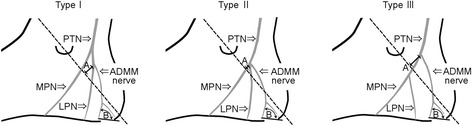
A schematic drawing of the local anatomical features of the nerve to the abductor digiti minimi muscle (*ADMM nerve*). The *dotted line* indicates the malleolar–calcaneal axis. *A*, the distance from the branching position of the ADMM nerve to the malleolar–calcaneal axis. *B*, the angle at the piercing point between the medial process of the calcaneal tuberosity and the branching position of the cutaneous branch of the ADMM nerve. *LPN* lateral plantar nerve, *MPN* medial plantar nerve, *PTN* posterior tibial nerve

The branching pattern of the ADMM nerve was classified into 3 types: Type I, directly from the lateral plantar nerve; Type II, from the bifurcation of the posterior tibial nerve; and Type III, directly from the posterior tibial nerve (Fig. 1).

It is known that the lateral plantar nerve branches off from the posterior tibial nerve under the flexor retinaculum and that the branching position is within 2 cm of the malleolar–calcaneal axis between the top of the medial malleolus and the medial process of the calcaneus [6]. Accordingly, the distance from the branching position of the ADMM nerve to the malleolar–calcaneal axis (A in Fig. 1) was measured with a digital caliper (Sinwa, Model 19979, JPN) and the median value of 3 measurements (in millimeter) was obtained. At the time of measurements, all feet were fixed in the anatomical position after the Achilles tendon was cut, and the plantar aponeurosis and abductor hallusis muscle were removed. The superior side of the malleolar–calcaneal axis was defined as the “plus” direction, and the inferior side was defined as the “minus” direction.

At the piercing point in the ADMM, the angle between the medial process of the calcaneal tuberosity and the branching position of the cutaneous branch of the ADMM nerve (B in Fig. 1) was measured with a digital goniometer (Sinwa, Model SA-5468, JPN) and the median value of 3 measurements was obtained.

All these measurements were performed 3 times in each foot after the ankle joint was fixed in the anatomical position, and the medians were calculated. Sex differences were evaluated using Student’s *t-*test and the *χ*^2^ test. Differences were considered significant if the *P* value was less than 0.05.

## Results

**Fig. 2:**
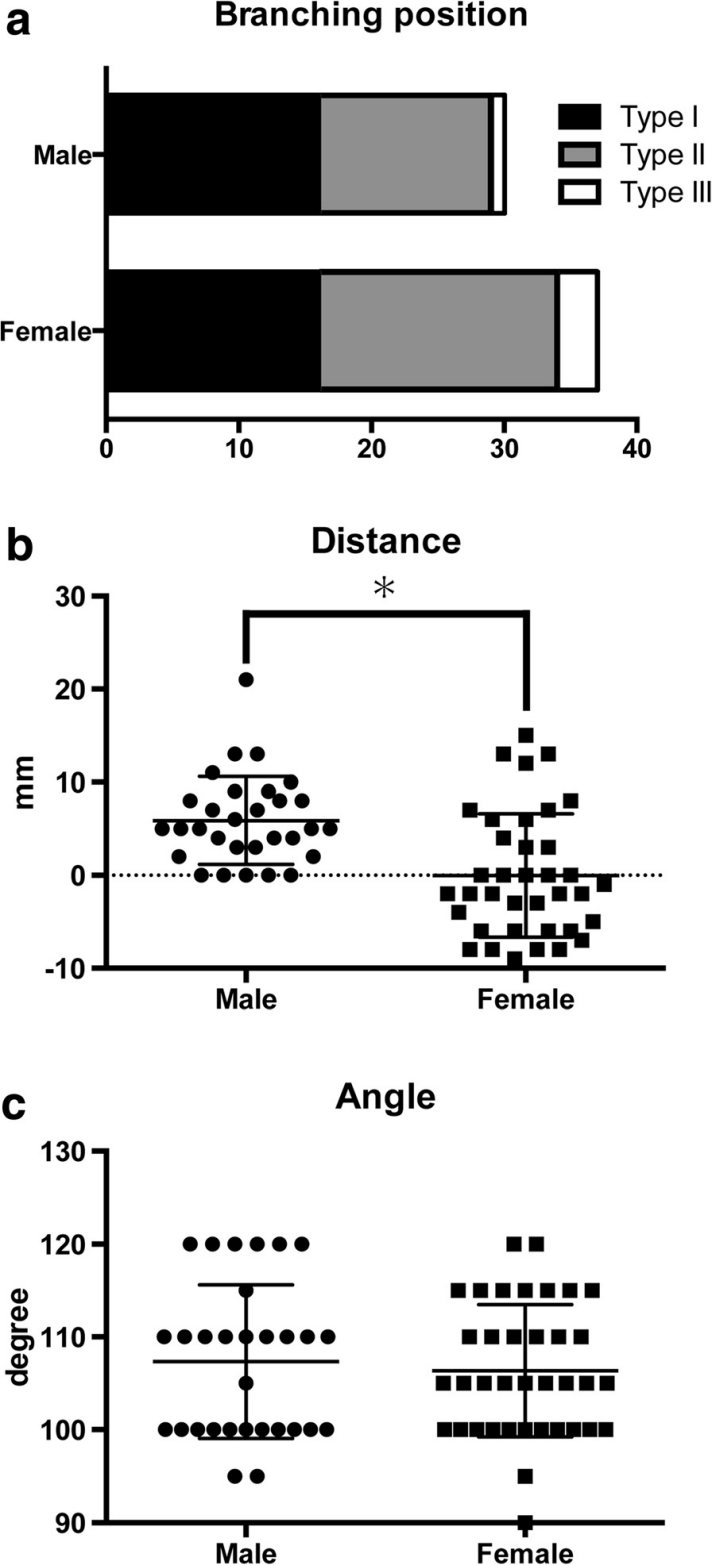
Statistical analysis of data from males and females. **a** Branching position of abductor digiti minimi muscle (ADMM) nerve. **b** The distance from the branching position of ADMM nerve to malleolar-calcaneal axis. **c** The angle at the piercing point to ADMM. *Asterisks* indicate that the *P* values were less than 0.0001

None of the feet had ADMM atrophy, myofascial adhesion, or a plantar heel spur. All ADMM nerves branched under the flexor retinaculum. The branching pattern of the ADMM nerves was Type I in 32 ft (48 %), Type II in 31 ft (46 %), and Type III in 4 ft (6 %). In males, the branching pattern was Type I in 16 ft (53 %), Type II in 13 ft (43 %), and Type III in 1 ft (3 %). In females, the branching pattern was Type I in 16 ft (43 %), Type II in 18 ft (49 %), and Type III in 3 ft (1 %). There were no significant differences in the branching pattern of the ADMM nerve between male and female feet (Fig. 2a, *P* = .58).

**Fig. 3:**
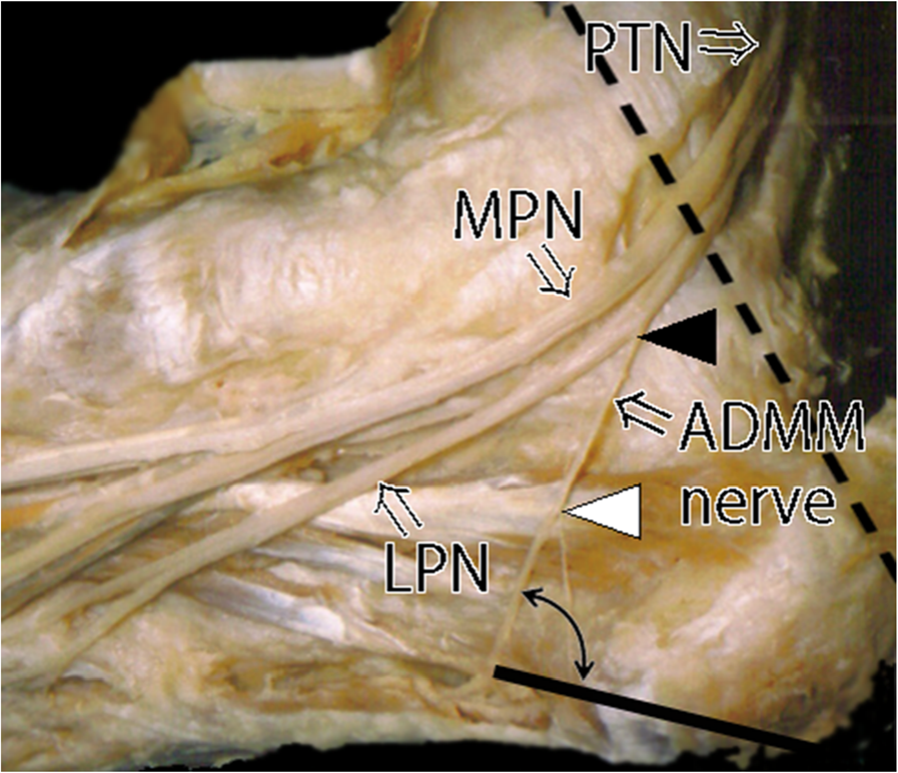
Gross anatomical findings in a female foot (No. 12, *right*) in which the nerve to the abductor digiti minimi muscle (*ADMM nerve*) branched below the malleolar–calcaneal axis. The *dotted line* indicates the malleolar–calcaneal axis. The *black arrowhead* indicates the branching position of the ADMM nerve. The *white arrowhead* indicates the branching position of the cutaneous branch. The two directional *arrows* indicate the angle at the piercing point in the ADMM between the medial process of the calcaneal tuberosity and whitehead. *LPN* lateral plantar nerve, *MPN* medial plantar nerve, *PTN* posterior tibial nerve

The branching positions of the ADMM nerve were superior to the malleolar–calcaneal axis (direction: plus) in 37 ft (55 %), on the malleolar–calcaneal axis (direction: zero) in 10 ft (15 %), and inferior to the malleolar–calcaneal axis (direction: minus) in 20 ft (30 %; Fig. 2b). There was no case among male feet in which the ADMM nerve branched inferior to the malleolar–calcaneal axis. On the other hand, this pattern was observed in 19 or 37 female feet (51 %) (Figs. 2b and 3). The distance from the malleolar–calcaneal axis to the branching position of the ADMM nerve was 5.9 ± 4.7 cm in male feet and 0.0 ± 6.6 cm in female feet. There was a statistically significant difference in the distance from the branching position of the ADMM nerve to the malleolar–calcaneal axis between male and female feet (Fig. 2b, *P* < .0001).

The angle at the piercing point in the ADMM was 107.3 ± 8.3° in males and 106.4 ± 7.1° in females. There was no significant difference in this angle between male and female feet (Fig. 2c, *P* = .60).

## Discussion

In this study, we assessed sex differences in the branching pattern, position, and angle of the ADMM nerve in cadaveric feet. Because the diameter of the ADMM nerve at the branching position is approximately 2–3 mm, it is difficult to evaluate these differences using MRI or ultrasonography. To the best of our knowledge, this is the first report on sex differences in the branching pattern, position, and angle of the ADMM nerve.

Damage to the ADMM nerve was first reported in 1940 by Roegholt^18^ and has subsequently been confirmed by numerous authors [1, 4, 8, 11–18]. The damage has been postulated to occur in one of two locations: either at the point where the nerve changes direction at the inferior margin of the abductor halluces, where it is compressed between the abductor hallucis and the medial side of the quadratus plantae, or slightly more distal, where the nerve passes anterior to the medial process of the calcaneal tuberosity [4, 14]. The present study could not find significant sex differences in either the branching pattern or the angle of ADMM nerves in these locations. It has been reported that the scar tissue after plantar fasciitis causes damage to the ADMM nerve [12]; however, sex differences in the incidence of plantar fasciitis have not been reported. The incidence of a plantar heel spur is higher in females [14], and Roegholt [19] reported some cases where a plantar heel spur caused damage to the ADMM nerve. On the other hand, Tanz [20] argued that the presence of a plantar heel spur is not related to heel pain. Therefore, the extent to which a plantar heel spur affects the prevalence of ADMM nerve damage or ADMM atrophy remains unclear. To determine this relationship, a retrospective study involving MRI data is being planned.

**Fig. 4:**
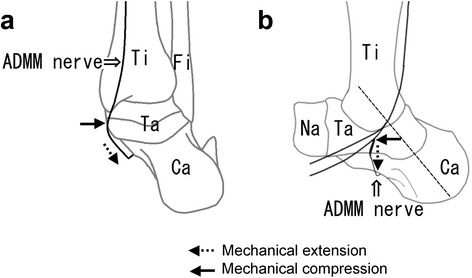
A schematic drawing of the mechanical extension and compression of the nerve to the abductor digiti minimi muscle (*ADMM nerve*). **a** The posterior view. **b** The medial view. *Ca* calcaneus, *Fi* fibula, *Ta* talus, *Ti* tibia, *Na* navicular bone

The present study showed a significant sex difference in the branching position of the ADMM nerve, but not in the branching pattern. There was no case among male feet in which the ADMM nerve branched inferior to the malleolar–calcaneal axis, whereas this pattern was observed in 19 of 37 female feet (51 %). It is well recognized that females are more variable than males with regard to any morphology. With regard to sex differences in the lower extremities, the Q angle is greater in females than in males. Nguyen et al. [21] reported that sex differences are linked to the characteristics of lower extremity alignment and hip muscle activation, such that females have a greater pelvic angle, femoral anteversion, quadriceps angle, tibiofemoral angle, and genu recurvatum compared with males. Steinberg [22] demonstrated that the Q angle and excessive foot pronation are strongly correlated with hallux valgus. In fact, hallux valgus occurs more often in females than in males. It can be hypothesized that sex differences in lower extremity alignment are also linked to the higher prevalence of ADMM nerve damage in females. This is because excessive foot pronation overloads the ADMM nerve with an increased Q angle, and consequently, mechanical extension and compression are applied to the ADMM nerve, as shown in Fig. 4a. In particular, in cases where the ADMM nerve branches below the malleolar–calcaneal axis, the mechanical extension and compression must increase and should damage the ADMM nerve, as shown in Fig. 4b. Further research should focus on mechanical evaluation of ADMM nerve damage from the standpoint of sex differences, including the differences in footwear, e.g., high heels. Besides, since hormonal sex differences may contribute to ADMM nerve damage, such as de Quervain disease and carpal tunnel syndrome, these differences should also be explored.

## Conclusions

In conclusion, the present study showed that the ADMM nerve branched below the malleolar–calcaneal axis in half of female feet and none of the male feet. Although the reason for the more frequent occurrence of damage to the ADMM nerve in females than in males has not be elucidated sufficiently, this difference may be a cause of the female predominance of nerve damage. Further research is warranted to identify the mechanisms underlying ADMM nerve damage.
